# Supplementary material for: Protocol to measure and analyze protein interactions in mammalian cells using bioluminescence resonance energy transfer
Source: STAR Protoc. 2024 Sep 28;5(4):103348. doi: 10.1016/j.xpro.2024.103348 (PMC11470631; doi:10.1016/j.xpro.2024.103348)
Supplement: Document S1. Tables S1 and S2 [file mmc1.pdf]

Supporting Information

Supplementary Tables

Table S1. Example amounts of transfected siRNA a day before transfection of BRET-biosensor and donor-only (BRET-control) plasmids for donor saturation-titration BRET experiments, related to Part 1. "A" refers to the GFP2-tagged acceptor construct (GFP2-K-RasG12V) and "D" to the RLuc8-tagged donor construct (RLuc8-K-RasG12V). The pcDNA3.1-plasmid is used as empty vector to top up the transfected DNA amount to the same total per well.

siRNA	well number	A/D ratio		plasmid amounts/ ng			volume/ µL from 100 ng/ µL plasmid stock			siRNA/ nM*
		A	D	A	D	empty vector	A	D	empty vector	
FNTA siRNA	1	1	1	25	25	975	0.25	0.25	9.75	100
	2	4	1	100	25	900	1	0.25	9	100
	3	8	1	200	25	800	2	0.25	8	100
	4	12	1	300	25	700	3	0.25	7	100
	5	16	1	400	25	600	4	0.25	6	100
	6	24	1	600	25	400	6	0.25	4	100
	7	32	1	800	25	200	8	0.25	2	100
	8	40	1	1000	25	0	10	0.25	0	100
	9 BRET-control	0	4	0	1	9	0	1	9	0
negative control siRNA	10	1	1	25	25	975	0.25	0.25	9.75	100
	11	4	1	100	25	900	1	0.25	9	100
	12	8	1	200	25	800	2	0.25	8	100
	13	12	1	300	25	700	3	0.25	7	100
	14	16	1	400	25	600	4	0.25	6	100
	15	24	1	600	25	400	6	0.25	4	100
	16	32	1	800	25	200	8	0.25	2	100
	17	40	1	1000	25	0	10	0.25	0	100
	18 BRET-control	0	4	0	1	9	0	1	9	0

* The siRNA e.g. targeting the gene *FNTA* is transfected using Lipofectamine RNAiMAX a day before the transfection of BRET-biosensor constructs and the donor-only control which is not transfected with any siRNA but only receives RNAiMAX. Growth medium containing siRNA and RNA-transfection reagent needs to be removed before transfecting plasmids. Subsequently, cells are DNA transfected as described in **part 2**. Two saturation-titration curves can then be obtained and compared, one transfected with *FNTA* siRNA and the other with negative control siRNA.

Table S2. Example amounts of transfected BRET-biosensor and donor-only (BRET-control) plasmids for donor saturation-titration BRET experiments with drug treatment, related to Part 2. “A” refers to the GFP2-tagged acceptor construct (GFP2-K-RasG12V) and “D” to the RLuc8-tagged donor construct (RLuc8-K-RasG12V). The pcDNA3.1-plasmid is used as empty vector to top up the transfected DNA amount to the same total per well. Two 12-well plates are needed for the saturation-titration curves, one for the vehicle-control and one for the treatment with 5 µM mevastatin in 0.1% DMSO/ growth medium. For the Mevastatin treatment, prepare a 5 mM stock solution diluted in DMSO. Take two Falcon tubes containing each 9 mL of growth medium in which you add 9 µL of mevastatin in the first and 9 µL of DMSO in the second one, and vortex thoroughly to mix the medium with the compounds. Then, replace the 1 mL medium in each well with 1 mL from the corresponding DMSO or Mevastatin treatment.

treatment	well number	A/D ratio		volume/ µL from 100 ng/ µL plasmid stock			drug stock / µL**
		A	D	A	D	empty vector	
vehicle control	1	1	1	0.25	0.25	9.75	1
	2	4	1	1	0.25	9	1
	3	8	1	2	0.25	8	1
	4	12	1	3	0.25	7	1
	5	16	1	4	0.25	6	1
	6	24	1	6	0.25	4	1
	7	32	1	8	0.25	2	1
	8	40	1	10	0.25	0	1
	9 BRET-control	0	4	0	1	9	0
5 µM Mevastatin	10	1	1	0.25	0.25	9.75	1
	11	4	1	1	0.25	9	1
	12	8	1	2	0.25	8	1
	13	12	1	3	0.25	7	1
	14	16	1	4	0.25	6	1
	15	24	1	6	0.25	4	1
	16	32	1	8	0.25	2	1
	17	40	1	10	0.25	0	1
	18 BRET-control	0	4	0	1	9	0

** Drug treatment is done the day after the DNA transfection for a total of 24 hours.
